# The impact of xerostomia on oral-health-related quality of life among younger adults

**DOI:** 10.1186/1477-7525-4-86

**Published:** 2006-11-08

**Authors:** W Murray Thomson, Herenia P Lawrence, Jonathan M Broadbent, Richie Poulton

**Affiliations:** 1 Department of Oral Sciences, School of Dentistry, University of Otago, Dunedin, New Zealand; 2 Community Dentistry Discipline, Department of Biological and Diagnostic Sciences, Faculty of Dentistry, University of Toronto, Canada; 3 Department of Preventive and Social Medicine, School of Medicine, University of Otago, Dunedin, New Zealand

## Abstract

**Background:**

Recent research has suggested that chronic dry mouth affects the day-to-day lives of older people living in institutions. The condition has usually been considered to be a feature of old age, but recent work by our team produced the somewhat surprising finding that 10% of people in their early thirties are affected. This raises the issue of whether dry mouth is a trivial condition or a more substantial threat to quality of life among younger people. The objective of this study was to examine the association between xerostomia and oral-health-related quality of life among young adults while controlling for clinical oral health status and other potential confounding factors.

**Methods:**

Cross-sectional analysis of data from a longstanding prospective observational study of a Dunedin (New Zealand) birth cohort: clinical dental examinations and questionnaires were used at age 32. The main measures were xerostomia (the subjective feeling of dry mouth, measured with a single question) and oral-health-related quality of life (OHRQoL) measured using the short-form Oral Health Impact Profile (OHIP-14).

**Results:**

Of the 923 participants (48.9% female), one in ten were categorised as 'xerostomic', with no apparent gender difference. There was a strong association between xerostomia and OHRQoL (across all OHIP-14 domains) which persisted after multivariate analysis to control for clinical characteristics, gender, smoking status and personality characteristics (negative emotionality and positive emotionality).

**Conclusion:**

Xerostomia is not a trivial condition; it appears to have marked and consistent effects on sufferers' day-to-day lives.

## Background

Chronic dry mouth affects a substantial proportion of the population, with reported prevalence estimates from representative samples of older people ranging from 12% to 47% [1], and 10% recently reported for people in their early thirties [2], depending on which feature of dry mouth is being measured. While xerostomia is the subjective feeling of dry mouth, and is assessed by directly questioning individuals [3], salivary gland hypofunction (SGH) results in salivary output (flow rate) which is lower than normal; it is determined by sialometry [4]. Prescribed medications are the major recognised risk factor for dry mouth [5].

While dry mouth has been reported to affect important aspects of life such as speaking, the enjoyment and ingestion of food, and the wearing of dental prostheses [6,7], only recently has its relationship with oral-health-related quality of life (OHRQoL) been systematically investigated. Xerostomia was found to be associated with OHRQoL in a convenience sample of institutionalised older people in Toronto [8]; that study used two different OHRQoL scales (the General Oral Health Assessment Index, or GOHAI [9], and the short-form Oral Health Impact Profile, or OHIP-14 [10]), and the association with xerostomia was strong with either measure. A Swedish study [11] of dry mouth and OHRQoL among a small sample of institutionalised older people found similar associations. While these findings suggest that the impact of dry mouth extends beyond the oral cavity and into people's day-to-day lives, there is a need for confirmation of the relationship in a representative sample, and among younger adults who are living in the community. Moreover, as was pointed out by Locker [8], an increasing focus on patient-based outcomes in an environment of ever-greater demands on scarce oral health resources means that identification of the conditions with the most potential to compromise OHRQoL is a matter of some urgency. Furthermore, recent research [12] which identified an association between OHRQoL and negative affectivity (defined as a general disposition to experience subjective distress) has raised the issue of the extent to which personality characteristics modify the relationship between OHRQoL and the clinical characteristics which would be expected to affect it. Thus, an examination of that relationship should attempt to control for those traits.

The aim of this study was to examine the association between xerostomia and OHRQoL (as the dependent variable) among 32-year-old participants in a longstanding prospective cohort study.

## Methods

The Dunedin Multidisciplinary Health and Development Study is a longitudinal study of a birth cohort of children who were born at the Queen Mary Hospital, Dunedin, New Zealand between 1 April 1972 and 31 March 1973 [13]. The sample that formed the basis for the longitudinal study was 1037 children, and they were assessed within a month of their third birthdays. Collections of health and developmental data (including dental examinations) have been undertaken periodically since then, and the current study uses data collected from dental examinations at ages 26 and 32. The most recent assessment was at age 32, when 972 participants (96% of the surviving cohort) took part. Over 90% of the cohort self-identified as being of European origin. Ethical approval for the study was obtained from the Otago Ethics Committee.

At age 32, Study members were asked the question "How often does your mouth feel dry?" (response options 'Always' 'Frequently' 'Occasionally' or 'Never'). At the analysis stage, those who had responded 'Always' or 'Frequently' were designated as "xerostomic" [14].

The short-form Oral Health Impact Profile (OHIP-14) [10] was used at age 32. For each of the 14 items (Table 1), Study members were asked how often in the previous 4 weeks they had experienced the problem. Responses were coded as 'Very often' (scoring 4), 'Fairly often' (3), 'Occasionally' (2), 'Hardly ever' (1) or 'Never' (0). OHIP-14 scores were computed in two ways: first, an overall OHIP-14 score was calculated by summing responses over all 14 items; second, OHIP-14 subscale scores were calculated for each of the dimensions indicated in Table 1 by summing the response scores for the two corresponding items. Item weights were not used. The total OHIP-14 score and the subscale scores constitute measures of the 'severity' of adverse impacts caused by oral conditions and, as such, these measures used all response categories [15]. The prevalence of impacts was computed (at item, subscale and whole-scale level) by identifying individuals who experienced impacts 'Very often' or 'Fairly often'.

**Table 1:** Prevalence of OHIP-14 individual item impacts ('very often' or 'fairly often') by xerostomia status (brackets contain percentages)

**Item (and dimension in italics)**	**Xerostomic?**	
*Because of trouble with your teeth, mouth or dentures:*	No	Yes
*Functional limitation*		
Have you had trouble pronouncing any words?	9 (1.1)	5 (5.5)^a^
Have you felt that your sense of taste has worsened?	7 (0.8)	11 (12.1)^a^
*Physical pain*		
Have you had painful aching in your mouth?	27 (3.2)	12 (13.2)^a^
Have you found it uncomfortable to eat any foods?	38 (4.6)	14 (15.4)^a^
*Psychological discomfort*		
Have you been self-conscious?	61 (7.3)	23 (25.3)^a^
Have you felt tense?	23 (2.8)	13 (14.3)^a^
*Physical disability*		
Has your diet been unsatisfactory?	67 (8.1)	14 (15.4)^a^
Have you had to interrupt meals?	27 (3.2)	9 (9.9)^a^
*Psychological disability*		
Have you found it difficult to relax?	15 (1.8)	12 (13.2)^a^
Have you been a bit embarrassed?	25 (3.0)	18 (19.8)^a^
*Social disability*		
Have you been a bit irritable with other people?	15 (1.8)	12 (13.2)^a^
Have you had difficulty doing your usual jobs?	9 (1.1)	4 (4.4)^a^
*Handicap*		
Have you felt that life in general was less satisfying?	15 (1.8)	10 (11.0)^a^
Have you been totally unable to function?	6 (0.7)	5 (5.5)^a^

^a^P < 0.05

Personality traits were measured at age 26, when participants completed a 177-item modified version (Form NZ) of the multidimensional personality questionnaire (MPQ), a self-report personality instrument examining a broad range of individual differences in emotional and behavioural style [16,17]. The relative independence of the 10 MPQ subscales has been previously reported [18]. The subscales define the three superfactors of constraint, negative emotionality and positive emotionality. The constraint factor comprises the traditionalism, harm avoidance and control subscales. Individuals scoring highly within those tend to be restrained, cautious, and conventional, while low scorers are impulsive, fearless and sensation-seeking, and reject conventional strictures on their behaviour. The negative emotionality factor comprises the aggression, alienation and stress reaction subscales: high scorers tend to be easily stressed and harassed, and are prone to experiencing strong negative emotions (such as anxiety or anger). The positive emotionality factor comprises the wellbeing, social potency, achievement and social closeness subscales: individuals scoring highly within those tend to interact positively with their environment, and are ready to experience the positive emotions which arise from those interactions. Low scorers report fear of these pleasurable transactions, a low degree of self-efficacy (the belief that they can influence their environment), and are less likely to be happy. Previous developmental studies have shown that the personality traits measured using the MPQ are predictable from childhood, and reasonably stable from adolescence to adulthood [18, 19].

Dental examinations for caries and missing teeth were conducted by two calibrated examiners who examined approximately 50% of the dentally assessed Study members each. Before examination of each Study member, forms were adjusted to account for teeth that had been missing at the previous (age 26) assessments. An estimation of accumulated tooth loss due to caries by age 32 was obtained by observing the presence or absence of each tooth at 32, and ascertaining the reason for its absence by asking the Study member at that time (if the tooth had not been missing by age 26). Only those teeth which had been lost because of caries are included in estimations of tooth loss due to caries (or in the 'M' component of DMF scores). Teeth were examined for caries and restorations, with the buccal, lingual, distal, and mesial surfaces being scrutinised for anterior teeth (canines and incisors); the occlusal surface was included for posterior teeth (premolars and molars). Where a surface could not be visualised by the examiner (due to excessive calculus or being covered by gingival tissue), the surface was excluded from the examination and later analyses.

Periodontal measurements were made in all four quadrants. Three sites (mesiobuccal, buccal, and distolingual) per tooth were examined, and gingival recession (GR, the distance in millimetres from the cemento-enamel junction to the gingival margin) and probing depth (PD, the distance from the tip of the probe to the gingival margin) were recorded, using a National Institute of Dental and Craniofacial Research periodontal probe. Periodontal data were not recorded from third molars or carious retained roots. Midbuccal measurements for molars were made at the midpoint of the mesial root. All measurements were rounded down to the nearest whole millimetre at the time of recording. Where the gingival margin was situated more that 1 mm coronally to the cemento-enamel junction, a negative value for GR was recorded. Periodontal measurements were not conducted on Study members who reported any contraindication to periodontal probing. The combined attachment loss (CAL) for each site was computed by summing GR and PD. Current smokers at age 32 were identified by a positive response to the question "Have you smoked every day for one month or more of the previous 12 months?".

Chi-square tests were used to examine the statistical significance of differences observed with categorical dependent variables (such as the prevalence of impacts). Associations between xerostomia and mean overall and subscale OHRQoL scores were tested for statistical significance using the Mann-Whitney U-test because of the skewed distribution of the latter. Poisson regression modelling was used to examine the association between xerostomia and total OHIP score while controlling for clinical oral health characteristics, gender, smoking status and MPQ superfactor scores (using a median split for the latter).

## Results

Xerostomia, dental examination and OHIP-14 data were available for 923 Study members, and all subsequent analyses are based on that number of participants (of whom 451 were female). In response to the xerostomia question, 196 (21.2%) answered 'Never', 636 (68.9%) 'Occasionally', 81 (8.8%) 'Frequently', and 10 (1.1%) 'Always'. Of the 91 (9.9%) thus categorised as 'xerostomic', 46 (50.5%) were female.

Data on the prevalence of individual OHIP item impacts 'very often' or 'fairly often' are presented in Table 1. All items showed statistically-significant associations with xerostomia. Among the xerostomic respondents, being self-conscious was the most prevalent impact (approximately one in four), followed by being embarrassed (approximately one in five) and discomfort when eating (approximately one in six). Every OHIP dimension had at least one item which was associated with xerostomia.

Data on the prevalence of OHIP-14 subscale impacts by xerostomia status are presented in Table 2. Almost half of those with xerostomia (but only about one in five of the remainder) reported one or more impacts occurring 'very often' or 'fairly often', and the former had over three times the odds of doing so. Similar differences were observed with respect to all dimensions.

**Table 2:** Prevalence of any overall or subscale OHIP-14 impacts ('very often' or 'fairly often') by xerostomia status

	**Xerostomic?**		
**Dimension**	**No (%)**	**Yes (%)**	**Unadjusted odds ratio (95% CI)**
Any OHIP-14 impact	171 (20.6)	44 (48.4)^a^	3.6 (2.3, 5.6)
Functional limitation	15 (1.8)	14 (15.4)^a^	9.9 (4.6, 21.3)
Physical pain	46 (5.5)	19 (20.9)^a^	4.5 (2.5, 8.1)
Psychological discomfort	67 (8.1)	27 (29.7)^a^	4.8 (2.9, 8.1)
Physical disability	82 (9.9)	16 (17.6)^b^	2.0 (1.1, 3.5)
Psychological disability	37 (4.4)	22 (24.2)^a^	6.9 (3.8, 12.3)
Social disability	21 (2.5)	12 (13.2)^a^	5.9 (2.8, 12.4)
Handicap	16 (1.9)	10 (11.0)^a^	6.3 (2.8, 14.3)

^a^P < 0.0001

^b^P < 0.05

Mean overall and subscale OHIP-14 scores are presented by xerostomia status in Table 3. Those with xerostomia had higher overall and subscale scores on average, with all differences being statistically significant at the P < 0.001 level.

**Table 3:** Mean OHIP-14 combined and subscale scores, by xerostomia status (standard deviation in brackets)

	**Xerostomic?**	
**Dimension**	**No**	**Yes**
Overall OHIP-14 score	7.19 (7.27)	15.22 (10.89)^a^
Functional limitation	0.52 (0.97)	1.53 (1.76)^a^
Physical pain	1.59 (1.72)	2.79 (1.89)^a^
Psychological discomfort	1.29 (1.64)	2.74 (2.26)^a^
Physical disability	1.42 (1.62)	2.45 (1.97)^a^
Psychological disability	1.16 (1.45)	2.49 (2.17)^a^
Social disability	0.73 (1.25)	1.84 (2.04)^a^
Handicap	0.47 (1.01)	1.38 (1.87)^a^

^a^P < 0.001

The mean number of decayed surfaces (DS) at age 32 was 2.3 (sd, 4.8; range 0 to 42), and the mean number of teeth missing by that age due to dental caries was 0.6 (sd, 1.6; range 0 to 17). Two or more sites with 4+mm CAL were observed in 181 individuals (19.6%). Mean superfactor scores for the MPQ were: positive emotionality 269.3 (sd, 54.4; range 99.0 to 382.6); negative emotionality 80.9 (sd, 49.7; range 0.0 to 250.8); and constraint 187.2 (sd, 47.7; range 27.2 to 294.7). There were 305 current smokers (33.0%) at age 32. MPQ data were not available for 8 individuals. Associations between OHIP prevalence, xerostomia prevalence and these oral disease and MPQ superfactor scores are presented in Table 4. Xerostomia prevalence was associated with the number of untreated carious surfaces, the number of teeth lost due to caries, current smoking, and the negative emotionality MPQ superfactor. The prevalence of one or more OHIP impacts was associated with the number of untreated carious surfaces, the number of teeth lost due to caries, having periodontal disease, current smoking, and higher scores on the negative emotionality MPQ superfactor but lower scores on that for positive emotionality.

**Table 4:** Oral disease, smoking and personality characteristics by xerostomia status and OHIP impact prevalence at 32

	**Xerostomic?**		**Any OHIP-14 impact?**	
	**No**	**Yes**	**No**	**Yes**
No of decayed surfaces (DS) at 32 (sd)	2.2 (4.4)	4.0 (7.2)^a^	1.7 (3.4)	4.6 (7.3)^a^
No of teeth missing (due to caries) by 32 (sd)	0.5 (1.5)	1.0 (2.5)^a^	0.4 (1.3)	1.2 (2.2)^a^
Case of periodontal disease (2+ sites with 4+mm CAL) at 32 (%)	159 (19.1)	22 (24.2)	116 (16.4)	65 (30.2)^a^
Number of smokers at 32 (%)	265 (31.9)	40 (44.0)^a^	192 (27.1)	113 (52.6)^a^
Mean MPQ superfactor scores				
Positive emotionality (sd)	269.3 (55.0)	269.4 (48.7)	272.8 (53.5)	257.7 (56.0)^a^
Negative emotionality (sd)	78.2 (49.0)	105.9 (49.6)^a^	73.7 (45.7)	104.7 (54.9)^a^
Constraint (sd)	188.0 (48.1)	180.1 (43.2)	188.9 (47.7)	181.6 (47.4)

^a^P < 0.05

The strong association between xerostomia and the overall OHIP score remained after Poisson regression was used to control for clinical characteristics, gender, current smoking, negative emotionality and constraint (Table 5). As well as the xerostomics and those with more missing teeth or untreated caries, current smokers had higher OHIP scores (on average), as did those who scored more highly on negative emotionality or lower on positive emotionality. The modelling procedure was repeated for each of the subscale scores and, in each case, there was a strong association, with xerostomics having significantly higher subscale scores than non-xerostomics (Table 6). Negative emotionality and positive emotionality were also significantly associated with each subscale score, with higher scorers on the former (and lower scorers on the latter) having greater impacts.

**Table 5:** Poisson regression model for total OHIP score (R^2 ^= 0.16)

	**IRR^a^**	**95% CI for IRR**	**P value**
Xerostomia	1.74	1.64, 1.84	<0.0001
Female gender	0.93	0.89, 0.98	0.003
No of decayed surfaces (DS) at 32	1.03	1.02, 1.03	<0.0001
No of teeth missing (due to caries) by 32	1.01	1.00, 1.02	0.015
Case of periodontal disease (2+ sites with 4+mm CAL at 32)	1.05	0.97, 1.14	NS
Current smoking	1.41	1.34, 1.48	<0.0001
MPQ Negative emotionality (score > median)	1.40	1.33, 1.47	<0.0001
MPQ Positive emotionality (score < median)	1.21	1.16, 1.27	NS

^a^Ratio of geometric means

**Table 6:** Summary of Poisson regression models for OHIP subscale scores

	**IRR for xerostomia cases^a^**	**Model R^2^**
Functional limitation	2.28 (1.87, 2.78)	0.11
Physical pain	1.46 (1.27, 1.67)	0.06
Psychological discomfort	1.70 (1.48, 1.96)	0.08
Physical disability	1.54 (1.33, 1.78)	0.05
Psychological disability	1.73 (1.49, 2.00)	0.08
Social disability	2.00 (1.68, 2.39)	0.10
Handicap	2.28 (1.85, 2.80)	0.12

^a^Adjusted for the same covariates used in Table 4; brackets contain 95% confidence interval

## Discussion

This study of xerostomia and OHRQoL in a representative sample of relatively young adults has found that the two are strongly associated, with clear differences between xerostomics and the remainder with respect to all examined domains: xerostomia among younger adults certainly does matter. The findings not only support those from a recent study of a convenience sample of institutionalised older people in Toronto [8], but they build on them by the use of a representative sample (enhancing generalisability), their focus on younger adults, the controlling of potentially confounding personality traits, and the close examination of the association across the various subscales of the OHIP-14.

While it might (at first glance) seem somewhat inappropriate to even be investigating xerostomia and its associations among younger adults, the condition's surprisingly high prevalence among 32-year-olds – together with its strong association with OHRQoL – provides more than enough justification for having done so in the Dunedin Study. It is anticipated that future data collection phases in that study will provide unique information on the natural history and associations of xerostomia as the cohort enters middle age.

An important methodological consideration is whether it was appropriate to use the OHIP with 32-year-olds, given that it was originally developed and validated (and evolved into a short-form measure) using older people [20]. However, a considerable amount of research has been undertaken since that original work, focusing on examining the measure's properties in different cultures, age groups and settings. Its recent validation among adolescents [21] means that its use with 32-year-olds should therefore not be an issue. It would perhaps have been useful to use another OHRQoL measure in order to confirm the current study's strong association between xerostomia and OHIP scores – particularly in view of Locker's use of the GOHAI [9] and the OHIP in the only previous study to have investigated the relationship – but this was not possible because of time constraints during the data collection. Moreover, it is likely that using the short-form OHIP has led to a degree of under-estimation of impact prevalence, and, if anything, the use of the 49-item OHIP might have led to stronger associations being observed.

The robust association between xerostomia and OHRQoL persisted despite our best efforts to attenuate it by controlling for indicators of poor clinical oral health (such as untreated caries, periodontal disease and incremental tooth loss), smoking status and personality characteristics. That there was a strong association of OHRQoL with negative emotionality and constraint scores supports the findings of Kressin et al [12], who observed a similar association with poor OHRQoL among older male US veterans. Those who score highly on negative emotionality tend to be easily stressed and harassed, and are prone to experiencing strong negative emotions (such as anxiety or anger); it comes as no surprise that such individuals were more likely to report not only poorer OHRQoL (other factors being equal) but also more severe symptoms of dry mouth. The association with positive emotionality was also robust and consistent, with those scoring lower on that MPQ superfactor being less likely to be happy with their lot, and therefore more likely to report poorer OHRQoL. The data in Table 4 strongly suggest that negative emotionality, being associated with both xerostomia and poor OHRQoL, is a confounder of the association between the two, and the decision to control for it in the multivariate models was the correct one. The persistence of a strong xerostomia-OHRQoL association after controlling for possible confounders supports the assertion that xerostomia compromises oral-health-related quality of life.

## Conclusion

Chronic dry mouth (xerostomia) is strongly and independently associated with poorer oral-health-related quality of life among 32-year-olds. It is apparent from this study and earlier work [8] that xerostomia is not a trivial condition for anyone, whether they are relatively healthy younger adults or institutionalised older people. It is therefore appropriate to call for more intensive, systematic research efforts directed at clarifying the causes and natural history of the condition, together with investigation of the most appropriate preventive approaches and therapeutic interventions for the condition.

## Competing interests

The author(s) declare that they have no competing interests.

## Authors' contributions

WMT, HPL and JMB conducted the analyses, WMT and RP drafted the paper, and all authors made critical intellectual contributions to (and approved) the final manuscript.
